# A Longitudinal Study of Atrophy in Amnestic Mild Cognitive Impairment and Normal Aging Revealed by Cortical Thickness

**DOI:** 10.1371/journal.pone.0048973

**Published:** 2012-11-02

**Authors:** Zhijun Yao, Bin Hu, Chuanjiang Liang, Lina Zhao, Mike Jackson, the Alzheimer's Disease Neuroimaging Initiative

**Affiliations:** 1 School of Information Science and Engineering, Lanzhou University, Lanzhou, China; 2 Birmingham City Business School, Birmingham City University, Perry Barr, Birmingham, United Kingdom; 3 School of Computing, Telecomminications and Networks, Birmingham City University, Birmingham, United Kingdom; Institution of Automation, CAS, China

## Abstract

In recent years, amnestic mild cognitive impairment (aMCI) has attracted significant attention as an indicator of high risk for Alzheimer's disease. An understanding of the pathology of aMCI may benefit the development of effective clinical treatments for dementia. In this work, we measured the cortical thickness of 109 aMCI subjects and 99 normal controls (NC) twice over two years. The longitudinal changes and the cross-sectional differences between the two types of participants were explored using the vertex thickness values. The thickness of the cortex in aMCI was found significantly reduced in both longitudinal and between-group comparisons, mainly in the temporal lobe, superolateral parietal lobe and some regions of the frontal cortices. Compared to NC, the aMCI showed a significantly high atrophy rate in the left lateral temporal lobe and left parahippocampal gyrus over two years. Additionally, a significant positive correlation between brain atrophy and the decline of Mini-Mental State Examination (MMSE) scores was also found in the left superior and left middle temporal gyrus in aMCI. These findings demonstrated specific longitudinal spatial patterns of cortical atrophy in aMCI and NC. The higher atrophy rate in aMCI might be responsible for the accelerated functional decline in the aMCI progression process.

## Introduction

With the increasing size of the aging population, cognitive impairment has become an intractable problem which threatens the activities of daily life. Dementia, a serious form of cognitive impairment, reduces the ability to learn, communicate, reason, retain and recall past experience and may eventually trigger death [1], [2]. Mild cognitive impairment (MCI) is widely considered to be the clinical transition stage between normal aging and dementia [3]. Recent clinical studies have shown that patients with MCI have a significantly greater likelihood of progressing to Alzheimer's disease (AD) compared to healthy elderly subjects, with rates of progression estimated 6%∼25% per year [4]. However, MCI is a heterogeneous condition, i.e., not all MCI subjects develop dementia even after several years of follow-up. aMCI is a subtype in which subjects show early memory impairment, either with or without impairment of other cognitive domains, but do not fulfill the criteria for dementia [4]. A previous study showed approximately 80% of aMCI subjects will progress to a diagnosis of AD after a six years clinical follow-up [5]. The aMCI construct is therefore useful because it offers opportunities for relatively early diagnosis of AD.

MRI studies focus on patterns of cortical atrophy in aMCI in order to identify the earliest changes in the brain associated with AD, and predict which subjects will progress to a diagnosis of AD. Previous studies using the region of interest (ROI)-based MRI volumetric methods have found atrophy of medial temporal lobe structures in MCI [6], [7]. Atrophy in these structures including the hippocampus, parahippocampal gyrus and the amygdala etc. has been proved to differentiate cross sectionally among normal people, MCI and AD [8]. However, the anatomic ROIs need to be defined a priori and the manual methods to outline the ROIs are often laborious which makes it inconvenient for use in analyzing large samples. The almost universal global volumetric method to assess patterns of cortical atrophy is the voxel-based morphometry (VBM) method. VBM method can be used to assess differences in the regional concentration of grey matter between groups without the need for specifying ROIs apriori [9]. Studies applying VBM have found less grey matter volume in several regions including the medial temporal lobe, posterior cingulate gyrus and frontal lobe in aMCI and AD relative to controls [10], [11]. The more widespread and accelerated grey matter atrophy was also found in aMCI subjects who progress to AD within a fixed clinical follow-up time when compared to those who remain stable [12], [13]. Recently, the structural brain networks constructed using the grey matter volume in MCI and AD have been used to find abnormalities in the connectivity between different brain areas such as the bilateral parietal regions, middle temporal gyrus, etc. [14], [15]. Filippi et al. also found that patients with complex chronic neurodegenerative disorders such as aMCI experience a brain network breakdown [16]. The smoothing step in VBM, however, neglects the anatomical relationships across the folded cortical surface which might reduce the sensitivity to significant effects [17]. The measurement of cortical thickness has been recently proven to be both precise and sensitive in detecting alterations in cortical morphology [3]. It can provide a localized quantitative description of cortical atrophy (in millimeters) and enabling more precise measurement in deep sulci as a cortical sheet [18]. Cortical thickness analysis has been widely used to find the cortical thickness differences in normal controls, MCI, AD and frontotemporal dementia [6], [19]. Additionally, a recent study also utilized analysis of cortical thickness to examine differences between single-versus multiple-domain aMCI and predict the conversion from MCI to AD [20].

From the above discussions, we can see that most of the previous studies about aMCI focused on the structural changes between aMCI and normal people or aMCI and AD [12], [19]. Studies about longitudinal cortical changes in one fixed aMCI group are rare. There is also no report on the longitudinal differences of atrophy rate between aMCI and NC. The goal of this study therefore, was through the use of cortical thickness analysis to investigate the longitudinal spatial distribution of brain atrophy in a relatively large number of subjects with aMCI and NC. We compared the between-group differences of the cortical thickness at baseline time and two years later. Then we compared the cortical thickness differences between the baseline time and two years later in aMCI and NC respectively. The atrophy rate over two years between aMCI and NC was also compared. Finally, a correlation analysis was performed to explore the correlation between the decline of MMSE scores and cortical atrophy. The hypothesis was that the comparison between aMCI and NC would allow us to find the longitudinal spatial patterns of cortical atrophy in aMCI and NC. Moreover, the regions which showed higher atrophy rate in aMCI might be responsible for the neural functional decline.

## Materials and Methods

### Ethics Statement

For the purpose of this analysis we used ADNI subject data that was previously collected across 50 sites in the United States and Canada. Study subjects gave written informed consent at the time of enrollment for imaging and genetic sample collection and completed questionnaires approved by each participating site's Institutional Review Board (IRB), including Albany Medical College, Banner Alzheimer's Institute and Baylor College of Medicine etc. The complete list of ADNI sites' IRBs can be found at the link: http://adni.loni.ucla.edu/about/data-statistics/.

### Participants

All the subjects used in this study were obtained from the *Alzheimer's Disease Neuroimaging Initiative (ADNI)* database (www.loni.ucla.edu/ADNI). The primary goal of ADNI is to determine whether serial MRI, positron emission tomography (PET), other biological markers, and clinical and neuropsychological assessment can accurately measure the progression of aMCI and early AD.

This study was undertaken on a sample of 109 aMCI subjects (81 males/28 females) and 99 NC (50 males/49 females). Each participant was scanned twice, the baseline scan and again two years later. The exact time interval between two scans in aMCI is 25.1±0.5 months with range of 24.5–26.1 months while the interval is 25.4±0.8 months with range of 24.3–26.7 months in NC. The baseline scan data of aMCI subjects was named MCI_M1 group (age of 75±7 years with range of 55–88 years) while the NC was named NC_M1 group (age of 75±5 years with range of 50–88 years) and the follow-up scan was named MCI_M2 group and NC_M2 group respectively. The MMSE scores of MCI_M1 and MCI_M2 were 27.2±1.8 and 25.6±3.7 and NC_M1 and NC_M2 were 29.2±0.9 and 29.0±1.2.

All the normal controls were still normal aging and all the aMCI subjects recruited were still aMCI according to the criteria of ADNI database for NC and aMCI over two years. Criteria for the diagnostic of NC were 1) no active neurological or psychiatric disorders; 2) some subjects may have had ongoing medical problems yet the illnesses or their treatments did not interfere with cognitive function; 3) normal neurological exam; 4) were independently functioning community dwellers [13]. Criteria for the diagnosis of aMCI were those of Petersen et al. [4]: (1) memory complaint, preferably corroborated by an informant; (2) memory impairment for age; (3) essentially normal general cognitive function; (4) generally preserved activities of daily living; (5) not demented.

### MRI Imaging Acquisition

All anatomical high-resolution T1-weighted images of the whole brain were acquired sagittally on a 1.5-Tesla MRI scanner using a volumetric 3D MPRAGE (3-dimensional magnetization-prepared rapid acquisition gradient echo) sequence with the following parameters: slice thickness, 1.2 mm; echo time (TE), 3.61 ms; repetition time (TR), 3000 ms; flip angle, 8°; matrix size, 192×192; number of slices, 160; field of view, 1.25×1.25 mm.

### Image Preprocessing and Cortical Thickness Measurement

T1 images of all subjects were preprocessed with the volume and surface pipeline of FreeSurfer (http://surfer.nmr.mgh.harvard.edu/) [17]. The cortical thickness was obtained for each subject with the following steps: Firstly, images were corrected for intensity nonuniformity using N3 [21]. Secondly, images were registered via affine transformation to Montreal Neurological Institute space to strip the skull. Thirdly, binary white matter masks were generated using the segmentation information and corrected in areas that commonly produce topological defects. Fourthly, a triangle-based mesh of the white matter surface was then produced and expanded to get the pial surface. Fifthly, cortical thickness data was then determined for each subject by computing the mean of the two shortest distances between the white matter and pial surfaces. However, to compare the cortical thickness between two different groups, an average surface template was built using the curvature-based registration. Finally, all of the individual surfaces were aligned to this template and the distribution of original cortical thickness on the template was obtained using reparameterization.

### Statistical Analysis

Statistical analysis was performed with the vertices thickness values obtained in the data preprocessing.

To analyze whether any significant differences (p<0.05) in MMSE scores existed in the longitudinal observation, paired t-tests were carried out between the MMSE scores of two scans in aMCI and NC respectively.

Paired t-tests were also performed in the comparisons of MCI_M1 vs MCI_M2 and NC_M1 vs NC_M2 to test the significant longitudinal differences in cortical thickness. Multiple comparisons were taken into account for the vertex data using a false discovery rate (FDR) correction at a 0.01 level of significance [15].

Significant differences in cortical thickness between aMCI and NC were tested twice using the two-sample t-tests at homologous vertices while controlling for the effect of age and gender. The random field theory (RFT)-based cluster analysis was used to perform the multiple comparison correction in both comparisons of MCI_M1 vs NC_M1 and MCI_M2 vs NC_M2 [22]. We set a threshold for this analysis so that only contiguous vertex with a p value <0.001 was used to define clusters. Then a corrected cluster-wise p-value was obtained using the RFT. The significant level for clusters was set at p<0.05 after the multiple comparisons and only clusters with a minimum of 100 points were reported [17].

We also compared the atrophy rate of two years between aMCI and NC. At first, two matrixes of cortical thickness atrophy rate at every vertex were obtained through the cortical thickness of MCI_M1 minus those of MCI_M2 and then NC_M1 minus NC_M2. The data was then regressed to remove the effects of gender and age. Finally the two-sample t-tests were used to test for statistically significant differences in cortical thickness atrophy rate at homologous vertices between the aMCI and NC. The random field theory (RFT)-based cluster analysis was also used here to perform the multiple comparison correction. The significant level and the size for cluster were same as the setting above.

Finally, the Pearson correlation coefficients were used to explore whether there were some relationships between brain atrophy and neural functional decline. We did the same analysis for aMCI and NC separately. Initially we obtained the differences of cortical thickness and the differences of MMSE scores over two years. Then the Pearson correlation between the differences of cortical thickness and the differences of MMSE scores was performed for every vertex. To correct for multiple comparison, a FDR test was performed using a p value of 0.01 [15].

## Results

### Longitudinal changes of cortical thickness in aMCI and NC

We compared the cortical thickness of the two scans over two years at homologous vertices. Significant differences were found in both the MCI_M1/MCI_M2 and NC_M1/NC_M2 comparisons. In the groups two years later (MCI_M2 and NC_M2), cortical thickness on all the significantly different vertices is thinner than those in the baseline scan.

Figure?- shows the vertexby-vertex differences in cortical thickness between the MCI_M1 and MCI_M2. Table?/ only contains detailed information about atrophied regions which contain more than 2000 vertices with the total of 327684 vertices in the whole brain. The thickness changes were found to be quite widespread throughout the cortex especially in the left hemisphere. The atrophy mainly appeared in the frontal lobe, the parietal lobe and the temporal lobe whilst the cortical changes were also seen in the occipital lobe, the parahippocampal gyrus and the insula. In the frontal lobe, the most significant differences appeared in the precentral gyrus, the prefrontal cortex and mediainferior prefrontal area. In the parietal lobe, atrophied areas mainly included primary somatosensory cortex, somatosensory association cortex and some parts of Wernicke's area. Some degree of change could also be seen bilaterally on the superolateral temporal lobes.

**Figure 1:**
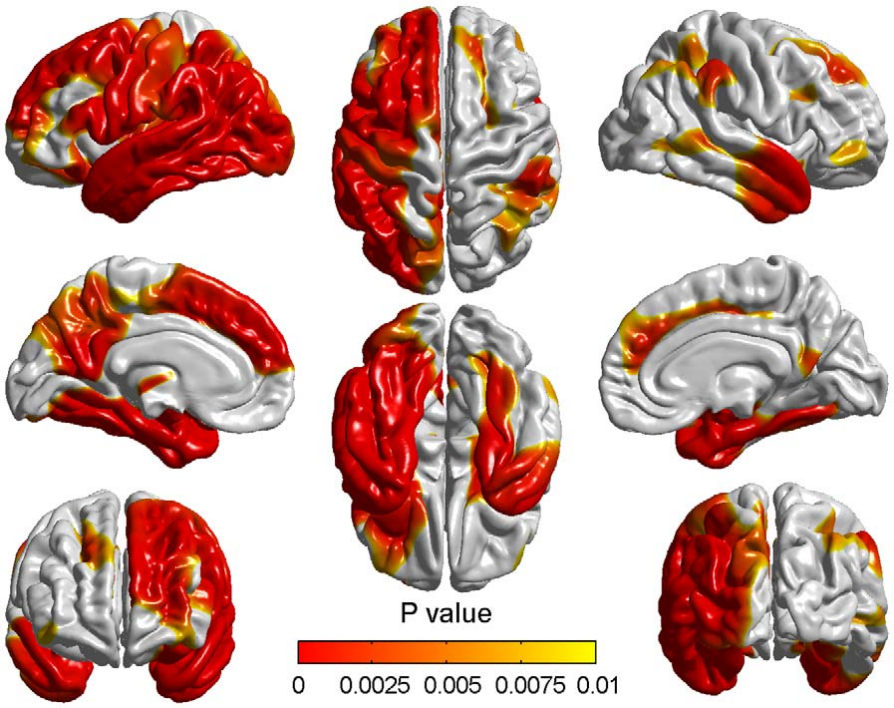
Differences of cortical thickness between the baseline and two years later in aMCI. The red/yellow showed the significantly different cortical thickness between the MCI_M1 and MCI_M2 groups. Only significantly reduced cortical thickness was found in the MCI_M2 group. The color bar indicated the vertex-wise p-value which ranged from 0 to 0.01 with the correction for multiple comparisons.

**Table 1:** Brain regions demonstrating significant difference in cortical thickness between MCI_M1 and MCI_M2 groups.

Region name	Vertices size	Maximum t value	Region name	Vertices size	Maximum t value
Precentral_L	8263	5.7524	Fusiform_R	2403	4.9885
Frontal_Sup_L	3936	5.7314	Postcentral_L	6854	4.9803
Frontal_Mid_L	6920	5.9494	Parietal_Sup_L	3312	4.9087
Frontal_Inf_Oper_L	2851	5.4962	Parietal_Inf_L	5543	6.3283
Frontal_Inf_Orb_L	2299	4.9262	Parietal_Inf_R	3435	4.3552
Rolandic_Oper_L	2083	5.4640	SupraMarginal_L	6458	6.0695
Supp_Motor_Area_L	4049	4.9487	SupraMarginal_R	2599	4.4784
Frontal_Sup_Medial_L	3705	5.5436	Angular_L	5220	7.4593
Frontal_Sup_Medial_R	2053	4.3258	Angular_R	2058	4.1197
Insula_L	6287	7.2763	Precuneus_L	6694	6.2796
ParaHippocampal_L	3174	8.7620	Temporal_Sup_L	6426	8.5361
ParaHippocampal_R	2441	6.0468	Temporal_Sup_R	2808	5.8012
Lingual_L	2076	7.1827	Temporal_Mid_L	5486	8.5511
Occipital_Mid_L	4788	6.9143	Temporal_Mid_R	3830	4.7491
Fusiform_L	3622	7.2777	Temporal_Inf_L	4906	8.2319

Note: vertices size is the number of significantly thin vertices included in the corresponding brain region. Maximum t value is the maximal t value obtained after the paired t-tests in the brain region.

The differences of cortical thickness between the NC_M1 and NC_M2 (Figure ) were not so generalized as those seen between the MCI_M1 and MCI_M2. The mainly atrophied regions are summarized in Table _. The most significant atrophy appeared in the left parahippocampal gyrus, right superior temporal gyrus and right superior temporopolar area as indicated by the areas shown in red. In contrast to those seen between MCIM1 and MCI_M2, changes between NC_M1 and NC_M2 were more prominent in the right hemisphere than the left.

**Figure 2:**
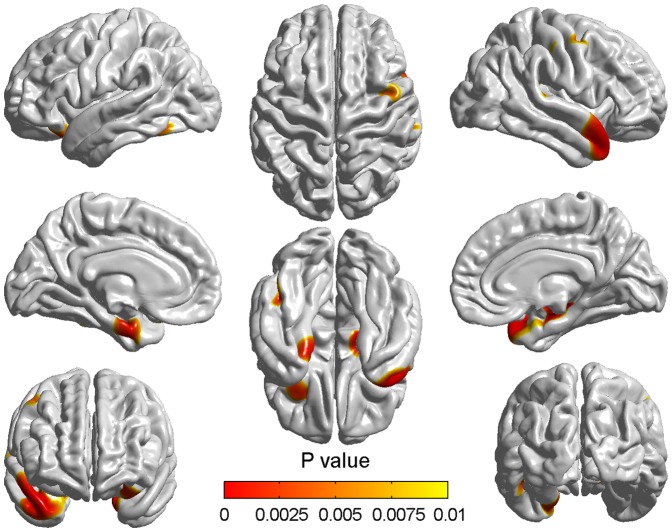
Differences of cortical thickness between the baseline and two years later in NC. The colorized areas indicated the different cortical thickness between the NC_M1 and NC_M2 groups. The range and degree of atrophy in NC_M2 were sight. The p values of the vertices indicated by the color bar were corrected using the FDR correction.

**Table 2:** Significant atrophy regions between NC_M1 and NC_M2 groups.

Region name	Vertices size	Maximum t value
Precentral_R	522	4.7192
Frontal_Inf_Orb_L	660	4.9231
Insula_R	335	5.7101
ParaHippocampal_L	762	5.1468
ParaHippocampal_R	293	5.1316
Fusiform_L	420	4.9273
SupraMarginal_R	302	4.5670
Temporal_Inf_L	260	4.9113
Temporal_Sup_R	1374	5.8042
Temporal_Pole_Sup_R	1136	5.8857

Note: The meaning of vertices size and maximum t value is same as the Table?.

### Between-group differences of cortical thickness at baseline time and two years later

At baseline time, aMCI showed significant cortical thinning compared to NC and no thicker brain regions were found in aMCI. Two clusters with thresholds of p<0.05 (RFT corrected) and cluster size>100 vertices were found (Figure?). These significantly different regions included parahippocampal gyrus, superior and middle temporopolar area, posterior cingulate gyrus, left temporal lobe, right middle and right inferior temporal gyrus, right superior orbital frontal cortex and right olfactory cortex (Table?).

**Figure 3:**
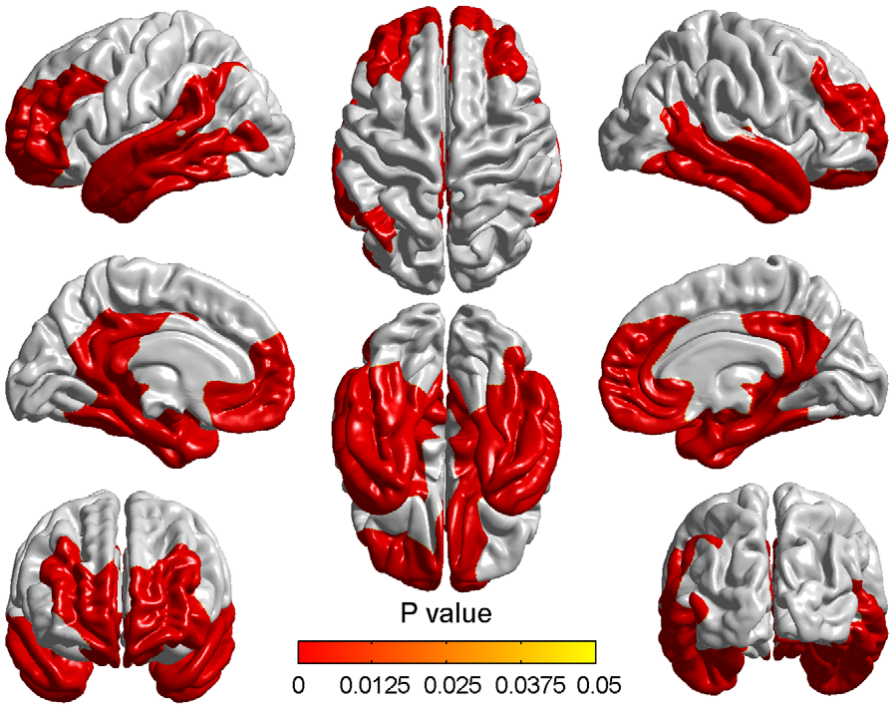
Cortical thinning in aMCI compared to NC at baseline time. The colorized areas indicated the brain regions with significantly reduced cortical thickness in aMCI compared with NC at baseline time with a correction for multiple comparisons (P<0.05, the cluster-based RFT correction).

**Table 3:** Brain regions demonstrating significantly reduced cortical thickness in aMCI at baseline.

Cluster no.	Region name	L/R	Peak MNI coordinates (x,y,z)	Peak t value	Number of vertices in cluster	RFT-corrected p value for cluster
1	Frontal_Sup_Orb	R	8,66,7	4.3465	58596	2.372e-08
	Olfactory	R	15,10,−18	4.2341		
	Cingulum_Post	R	5,−53,20	4.7801		
	ParaHippocampal	R	25,−11,−23	6.8813		
	Temporal_Pole_Sup	R	21,2,−36	5.9244		
	Temporal_Pole_Mid	R	27,−3,−38	6.2989		
	Temporal_Mid	R	48,0,−36	5.4139		
	Temporal_Inf	R	43,−2,−46	5.5002		
2	Cingulum_Post	L	−4,−47,12	4.8755	52428	2.372e-08
	ParaHippocampal	L	−19,−12,−23	7.1993		
	Temporal_Pole_Sup	L	−24,−1,−40	6.4533		
	Temporal_Pole_Mid	L	−30,−4,−36	6.6325		
	Temporal_Sup	L	−50,9,−37	5.3053		
	Temporal_Mid	L	−60,−19,−13	5.8685		
	Temporal_Inf	L	−31,−12,−37	6.3065		

Note: Peak t value is the maximum t value in the corresponding region. MNI is the Montreal Neurological Institute. Peak MNI coordinates is the coordinates of the peak vertex based on the MNI brain template.

Two years later, pronounced atrophy was found appearing in two significant clusters while comparing aMCI to NC (Figure?). Here, a multiple comparison correction was also performed with the same threshold. Compared with baseline time, the range and degree of the two clusters were wider and more pronounced in two years later. All the regions appeared at baseline remained after two years and the new involved regions were angular gyrus, left medial superior frontal gyrus, right fusiform gyrus, right superior temporal gyrus and right precuneus (Table?).

**Figure 4:**
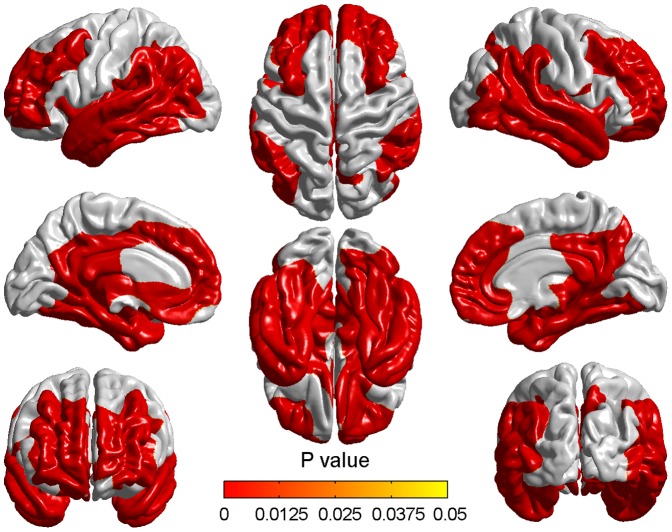
Cortical thinning in aMCI compared to NC two years later. Significant cortical thickness differences were found between aMCI and NC two years after the baseline time. The color bar indicated the cluster-wise p-value with the cluster-based RFT correction.

**Table 4:** Brain regions demonstrating significantly reduced cortical thickness in aMCI two years later.

Cluster no.	Region name	L/R	Peak MNI coordinates (x,y,z)	Peak t value	Number of vertices in cluster	RFT-corrected p value for cluster
1	Cingulum_Post	L	−2,−50,15	6.1246	96260	2.177e-08
	ParaHippocampal	L	−22,−14,−22	8.1999		
	Temporal_Pole_Sup	L	−24,−1,−40	7.4084		
	Temporal_Pole_Mid	L	−30,−4,−36	7.4984		
	Temporal_Sup	L	−51,0,−24	6.8052		
	Temporal_Mid	L	−60,−2,−22	7.2870		
	Temporal_Inf	L	−30,−11,−38	7.0859		
	Frontal_Sup_Medial	L	−20,60,22	4.5265		
	Angular	L	−49,−63,41	4.9026		
2	Frontal_Sup_Orb	R	10,66,9	4.4252	99789	2.177e-08
	Olfactory	R	17,8,−17	5.6264		
	Cingulum_Post	R	4,−53,19	6.4628		
	ParaHippocampal	R	25,−10,−24	8.0334		
	Temporal_Pole_Sup	R	21,2,−36	7.3326		
	Temporal_Pole_Mid	R	27,−3,−38	7.6556		
	Temporal_Mid	R	48,1,−36	6.9493		
	Temporal_Inf	R	43,−2,−46	7.1703		
	Temporal_Sup	R	50,−11,−17	6.2965		
	Fusiform	R	37,−21,−31	6.6532		
	Angular	R	45,−57,9	4.9855		
	Precuneus	R	4,−53,19	6.4641		

Note: The meanings of Peak t value and Peak MNI coordinates are the same as Table?.

### Differences of the atrophy rate in two years between aMCI and NC

Compared with the normal controls, the atrophy rate of aMCI appeared more pronounced. We found two significant clusters with threshold of p<0.05 (RFT corrected) and cluster size>100 (Figure?). The detailed information about the significantly different regions is presented in Table?. Cluster 1 included the superior temporal gyrus, the middle temporal gyrus, the temporopolar area, the inferior temporal and the insular cortex and cluster 2 only included the parahippocampal gyrus. All of these regions are located in the left hemisphere and the atrophy rate of all regions is more serious in the aMCI subjects.

**Figure 5:**
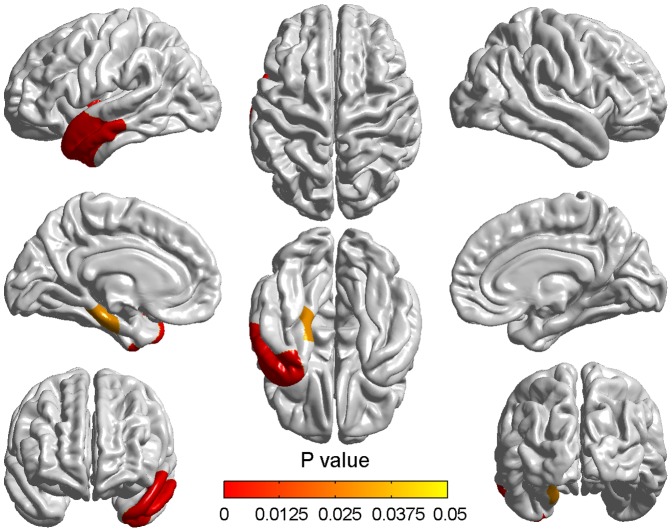
Differences of atrophy rate between aMCI and normal aging over two years. Differences of atrophy rate between aMCI subjects and normal controls were obtained using the random field theory (RFT)-based cluster analysis. The colorized areas represented the between-group differences of atrophy rate, which showed the more serious atrophy in aMCI over two years. The color bar indicated the cluster-wise p-value with the correction for multiple comparisons.

**Table 5:** Differences of atrophy rate between aMCI subjects and normal people.

Cluster no.	Region name	L/R	Peak MNI coordinates (x,y,z)	Peak t value	Number of vertices in cluster	RFT-corrected p value for cluster
1	Middle temporal gyrus	L	−53,1,−25	4.62	5913	0.00013351
	Insular cortex	L	−41,−6,0	3.57		
	Inferior temporal gyrus	L	−48,1,−37	4.08		
	Superior temporal gyrus	L	−51,2,−25	4.59		
	Temporopolar area	L	−49,9,−38	4.36		
2	Parahippocampal gyrus	L	−19,−26,−19	3.77	1227	0.031085

Note: The meanings of Peak t value and Peak MNI coordinates are same as Table?.

### Correlation analysis between the decline of MMSE scores and the cortical atrophy

For aMCI, the MMSE scores of MCI_M2 were significantly lower than those of MCI_M1 (p = 4.77e-06<0.05). There were no significant differences of MMSE scores between NC_M1 and NC_M2 (p = 0.23>0.05).

The correlation analysis showed no significant results in NC while a significant positive correlation in aMCI was found in brain regions largely corresponding to the regions demonstrating differences in atrophy rate between aMCI and NC (Figure?). The regions included left superior and left middle temporal gyrus, left superior and left middle temporopolar area (Table?).

**Figure 6:**
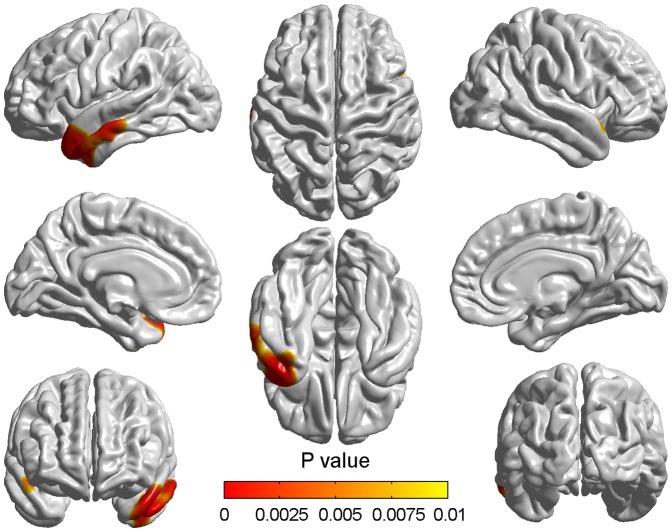
Correlation between the decline of MMSE scores and cortical atrophy in aMCI. The correlations at all significant vertices were positive and the color bar indicated the vertex-wise p-value with the FDR correction at a 0.01 level of significance.

**Table 6:** Brain regions with significant positive correlation between cortical atrophy and the decline of MMSE scores in aMCI.

Region name	Vertices size	Maximum R value	Region name	Vertices size	Maximum R value
Temporal_Sup_L	620	0.4887	Temporal_Mid_L	1666	0.4800
Temporal_Pole_Sup_L	582	0.4892	Temporal_Pole_Mid_L	429	0.4836

Note: Maximum R value is the maximal Pearson correlation coefficients in the corresponding brain region.

## Discussion

In this study, the vertex thickness values were used to explore the longitudinal patterns of cortical atrophy in aMCI and NC. The between-group differences of cortical thickness at two time points and atrophy rate over two years between aMCI and NC were compared. Additionally, the correlation between the decline of MMSE scores and the brain atrophy in aMCI and NC was also explored. The main findings of this study were the following four aspects: (i) Cortical thickness differences between baseline and two years later in aMCI and NC, respectively. For aMCI, cortical thinning appeared in the prefrontal gyrus, somatosensory cortex, Wernicke's area and superolateral temporal lobe after two years. In the second set of measurements for NC, atrophy mainly appeared in the left parahippocampal gyrus, right superior temporal gyrus and right superior temporopolar area. (ii) The between-group differences of cortical thickness at baseline time and two years later. At baseline, aMCI showed cortical thinning mainly in parahippocampal gyrus, temporopolar area, left temporal lobe, right middle and right inferior temporal gyrus. Two years later, aMCI demonstrated additional atrophy in angular gyrus, right fusiform gyrus and right superior temporal gyrus. (iii) Atrophy rate differences between aMCI and NC. Compared with NC, aMCI showed higher atrophy rate in left temporal lobe, left temporopolar area, left insula and left parahippocampal gyrus. (iv) Correlation between the decline of MMSE scores and cortical atrophy in aMCI and NC. Correlation analysis showed no significant results in NC while a significant positive correlation in aMCI was found in left superior and left middle temporal gyrus, left superior and left middle temporopolar area.

### Longitudinal spatial patterns of cortical atrophy in aMCI and NC

The longitudinal changes of cortical thickness in aMCI were quite widespread especially in the left hemisphere. The atrophy appeared in the precentral gyrus and prefrontal cortex showed a strong agreement with previous findings related to changes seen in MCI and AD [6], [19]. These regions have been implicated in planning complex cognitive behavior, planning and executing movements, personality expression, decision making and moderating social behavior and thus changes in these regions would not be unexpected [23]. Differences in the temporal lobe mainly located on the lateral left temporal lobe regions included the superior temporal gyrus, middle temporal gyrus and inferior temporal gyrus. These findings are also in accordance with neuropathological reports on both MCI and AD [24], [25]. Since the subjects we used were aMCI subjects and most of them are likely to develop AD, what we found could be explained by a previous finding that cortical differences in the lateral temporal lobe were seen between MCI and AD [26]. The changes in the parietal lobe are very pronounced particularly in the location of primary somatosensory cortex, somatosensory association cortex and some parts of Wernicke's area. These abnormalities of cerebral regions might be the reasons for the visuospatial and visual attention deficits presented in aMCI [27], [28].

Longitudinal atrophy in NC was relatively slight in both range and degree compared to aMCI. The atrophy mainly appeared in the left parahippocampal gyrus, right superior temporal gyrus and right superior temporopolar area. These findings showed a strong agreement with previous MRI findings related to changes seen in normal aging [29], [30]. The parahippocampal gyrus may be closely related to transmitting information from other areas into the limbic system and may be also responsible for visual memory encoding [31]. The superior temporal gyrus has also been considered to be a part of Wernicke's area and has been implicated in the understanding of written and spoken language and social perception [32]. All of these affected areas are polymodal and association cortices of the limbic system which might be thought to be related to many cognitive processes. Damage to these areas, therefore, might be in line with cognitive decline such as difficulties in learning strategics task and poor performance of refreshing information which are observed in the elderly [33], [34]. Additionally, though the longitudinal atrophy in NC was very slight, we found it was more prominent in the right hemisphere (Figure ). This asymmetric atrophy might be thought to support the previous viewpoint that the right hemisphere is more vulnerable to aging than the left hemisphere in the case of episodic memory retrieval [35].

### Differences of cortical thickness between aMCI and NC at two time points

Compared to NC, the aMCI showed significant cortical atrophy at two time points and the atrophy two years later was more pronounced than baseline time. All the atrophic regions existed at the baseline time remained two years later and the atrophy was more serious. Some regions such as angular gyrus, left medial superior frontal gyrus, right fusiform gyrus, right superior temporal gyrus and right precuneus showed significant atrophy after two years.

The most significant regions in both comparisons were parahippocampal gyrus, superior, middle and inferior temporal gyrus, superior and middle temporopolar area. Atrophy in these regions could be found in many previous studies and was considered to be responsible for the decline of corresponding functions in MCI such as language processing, scene recognition, recognition of known faces, sensation of sound and so on [24], [26], [36]. From the comparison of Table? and Table?, we found some atrophic regions which existed only two years later and this sequence of cerebral atrophy might indicate the spatial pattern of aMCI pathology. The pattern of AD pathology has been reported starting mainly in the hippocampus and entorhinal cortex, and subsequently spreading throughout most of the temporal lobe and posterior cingulate, finally involving extensive cortical regions, especially parietal, prefrontal, and orbitofrontal [36]-. Previous study also implied that the medial temporal region was the first one to be affected in MCI and as the disease progresses, posterior cingulate gyrus and temporoparietal association cortex were involved [37]. As the spatial pattern of brain atrophy in MCI is complex and highly variable, and to our knowledge the pattern of aMCI pathology changing over time has not been reported previously, the present results might be an insight towards understanding the sequence affected by aMCI.

### Differences of atrophy rate over two years between aMCI and NC

As we can see from above discussions, the atrophy over two years in aMCI appeared generalized in aMCI whilst there are also some slight cortical changes in normal aging. Thus, we could not ignore the effects of aging in spatial atrophy patterns in aMCI. To more intuitively reflect which regions were seriously affected in the progression of aMCI, we compared the atrophy rate between aMCI and NC. As Figure? shows, the atrophy rate of aMCI is higher in the superior temporal gyrus, the middle temporal gyrus, the temporopolar area, the inferior temporal, the insula and the parahippocampal gyrus compared with NC. We did not find any region which displayed more atrophy in NC than in aMCI. These regions might be responsible for the worse deficits of corresponding function in aMCI [38], [39].

The superior temporal gyrus has been considered to contain several important structures, such as the primary auditory cortex and Wernicke's area and be responsible for the sensation of sound and the processing of speech so that it can be understood as language [40]. This region has been suggested to be one of the areas in which the thinning was first observed in the progression of aMCI [41]. Atrophy of superior temporal gyrus seen in our results might explain the decreasing score of Boston Diagnostic Aphasia Battery comprehension in the aging-aMCI-AD continuum and predict the accelerated auditory comprehension deficits in aMCI [42]. The most significant differences of atrophy rate were involved in the middle temporal gyrus and the inferior temporal gyrus. Wang et al. also found that cortical thickness in the middle and inferior temporal gyri decreased in aMCI compared to control subjects [24]. Previous researches indicated that these two regions played an important role in the recognition of known faces and were responsible for visual word form perception [43]. In general, these two regions might be related to complex visual perception and the representation of complex object features [44]. Convit et al. reported that the combined middle and inferior temporal gyrus might be the first temporal lobe neocortical sites affected in AD and atrophy in these areas might herald the presence of future AD among non-demented individuals [29]. The greater atrophy of these two regions in aMCI might be associated with the higher level visual processing loss which has been well documented in patients with aMCI and AD [27], [38]. Parahippocampal gyrus is a region which is known to be related to memory encoding and retrieval especially in the encoding and recognition of scenes [31]. The atrophy of the parahippocampal gyrus can be seen in nearly every study about MCI and AD [24], [25], [45]. Subjects in this hypothesized transitional stage demonstrated diffuse amyloid in the neocortex and frequent neurofibrillary tangles in the medial temporal lobe such as the parahippocampal gyrus [39]. Thus, we hypothesize that further atrophy in parahippocampal gyrus might lead to the conversion from aMCI to AD [12]. Previous studies have reported the involvement of the insula in diverse functions usually linked to emotion or the regulation of the body's homeostasis, such as perception, motor control, self-awareness and cognitive functioning [46], [47]. Reduction of the cortical thickness and grey matter volume in insula has been noted in many previous aMCI studies [3], [24]. Nestor et al. also found that progressive non-fluent aphasia found in AD is associated with hypometabolism centred on the left anterior insula [48]. The greater atrophy of the left insula in aMCI found in this study might be thought to cause the decline of the corresponding physiological function in aMCI and the higher likelihood of developing dementia.

As depicted in Figure?, all of the significant brain regions were located in the left hemisphere. This might mean the left hemisphere is more likely to be affected by the pure aMCI pathology in the progression of aMCI. Actually, the left lateralized acceleration of grey matter degeneration has been reported in many previous studies about MCI and AD [3], [26]-. Meanwhile, the left hemisphere might be thought to be mainly responsible for the language comprehension, the logical analysis and the auditory verbal shortterm memory which were absent in aMCI [39], [42]. Our findings might provide further evidences to support the idea that the obvious asymmetrical atrophy appeared in aMCI [24], [25].

### Correlation between the decline of MMSE scores and cortical atrophy

To explore whether there was any relationship between the cortical atrophy and the degree of cognitive impairment, the Pearson correlation analyses and the MMSE scores were used. The results showed that in aMCI the atrophy in left superior and left middle temporal gyrus, left superior and left middle temporopolar area strongly correlated with the decline of MMSE scores (Figure?). Correlations in all of the four regions were positive and these regions had significantly higher atrophy rate in aMCI compared to NC (Figure?).

The relationship between MMSE and cortical atrophy has been noted in a few other studies and most of them focused on the hippocampal atrophy in MCI and AD [49], [50]. Our results were similar to previous study which found significant correlations between MMSE score and gray matter integrity existed in parahippocampal gyrus and temporal lobe [51]. The left lateralization might also be explicated by the theory that MMSE depended on the integrity of widely distributed cortical areas in both brain hemisphere with left-sided predominance.

In conclusion, our study found significant spatial patterns of cortical atrophy in aMCI and NC over two years. Compared to NC, aMCI showed the faster atrophy rate and a positive correlation between the regional atrophy and the neural functional decline over two years. The abnormal cerebral regions in aMCI especially the acceleratedly atrophic regions may deserve more attention for researchers in order to increase the understanding of the pathology of aMCI. This might help to find an effective clinical treatment to delay the conversion from aMCI to AD.
